# Comprehensive Pan-Cancer Analysis of ZNF668 Reveals the Prognostic and Immunological Significance of ZNF668

**DOI:** 10.3390/cimb47120997

**Published:** 2025-11-28

**Authors:** Xiaoyan Hu, Jiali Guo, Hua Zhong, Wenxin Huang, Size Chen, Canfeng He

**Affiliations:** 1 School of Clinical Medicine, Guangdong Pharmaceutical University, Guangzhou 510006, China; 2 Department of Immuno-Oncology, The First Affiliated Hospital of Guangdong Pharmaceutical University, Guangzhou 510080, China; 3 Department of Ultrasound, The First Affiliated Hospital of Guangdong Pharmaceutical University, Guangzhou 510080, China

**Keywords:** *ZNF668*, Pan-cancer analysis, TME, CAF, drugs

## Abstract

Transcription factors from the Zinc Finger Protein (ZFP) family are extensively implicated in tumorigenesis, yet the roles of many members, such as *ZNF668*, remain uncharacterized. This study presents a comprehensive pan-cancer analysis of *ZNF668*, investigating its expression profiles, genetic alterations, functional pathways, association with immune infiltration, and clinical correlations across cancer types from TCGA. Our pan-cancer analysis identifies *ZNF668* as a frequently overexpressed gene with significant diagnostic and prognostic value. Its overexpression, often driven by gene amplification, is linked to fundamental cellular processes such as RNA splicing and transcriptional regulation. Critically, *ZNF668* is implicated in promoting a state of adaptive immune resistance. While its expression positively correlates with the immunogenic MSI phenotype and suggests T-cell infiltration, this is likely offset by a dual immunosuppressive mechanism comprising a strong association with a cancer-associated fibroblast (CAF)-driven, T-cell-exhausted TME and a concurrent suppression of neutrophil recruitment. Furthermore, molecular docking identified Dasatinib as a potential *ZNF668* inhibitor. These findings establish *ZNF668* as a key regulator of CAF-mediated immune suppression, presenting it as a novel therapeutic target for restoring effective anti-tumor immunity.

## 1. Introduction

Cancer remains a leading cause of mortality worldwide, posing a significant global health challenge. According to the International Agency for Research on Cancer (IARC), 2022 saw an estimated 20 million new cancer diagnoses and 9.7 million deaths, with projections indicating new cases could surpass 35 million annually by 2050 [1]. Despite advances in conventional therapies like surgery, radiotherapy, and chemotherapy, their efficacy is often hampered by substantial adverse effects, acquired resistance, and high recurrence rates [2]. The emergence of immunotherapy, particularly immune checkpoint inhibitors, has marked a paradigm shift in oncology, offering durable clinical benefits for patients [3,4]. Nevertheless, the widespread clinical application of this transformative approach is hampered by the fact that a substantial proportion of patients exhibit either primary or acquired resistance, which remains a major clinical obstacle [5,6].

It is increasingly recognized that the tumor microenvironment (TME) is a pivotal determinant of cancer progression and therapeutic response [7]. This complex ecosystem, comprising tumor cells, immune cells, stromal cells, and the extracellular matrix, undermines therapeutic efficacy by promoting immunotherapy resistance through the formation of physical barriers and the release of suppressive signals [8,9]. The state of the tumor immune microenvironment is profoundly shaped by its underlying transcriptional landscape. Therefore, its dysregulation is rooted in the malfunctioning of a complex gene regulatory network that serves as the master blueprint for cellular identity and function. Therefore, elucidating the upstream master transcriptional regulators that govern this network has become a core scientific objective for deepening our understanding of the immunosuppressive microenvironment and identifying novel therapeutic targets to improve clinical outcomes.

Among these regulators, the Zinc Finger Protein (ZFP) family is of paramount importance. As the largest superfamily of transcription factors encoded by the human genome [10], ZFPs utilize zinc ions to form stable finger-like domains that mediate essential functions, including sequence-specific DNA binding, RNA recognition, and protein interactions [11,12]. Notably, ZFPs exhibit a striking functional duality in oncology, acting as both oncogenes and tumor suppressors [13]. For instance, oncogenic ZFPs such as *ZEB1*, *ZKSCAN3*, and ZFX drive malignancy by promoting proliferation, migration, epithelial-mesenchymal transition, and chemoresistance [14,15]. Conversely, tumor-suppressive ZFPs like *ZNF24* and *ZNF545* inhibit tumor growth by inducing cell cycle arrest and apoptosis [16,17].

While the ZFP family has been extensively researched, the specific role of its member, *ZNF668*, in cancer remains largely uncharacterized. Therefore, this study undertakes a comprehensive pan-cancer analysis to elucidate the functions of *ZNF668*. We conducted an integrated evaluation of its expression landscape, prognostic significance, genetic alterations, associated functional pathways, its relationship with tumor-infiltrating immune cells and pivotal immunoregulators. Collectively, this comprehensive analysis aims to elucidate the multifaceted role of *ZNF668* across various cancers, providing foundational insights into its potential as a prognostic indicator and its relevance in the tumor immune microenvironment.

## 2. Materials and Methods

### 2.1. ZNF668 Expression and Subcellular Localization Analysis

We analyzed the expression and localization of *ZNF668* using public databases. Pan-cancer gene expression data for normal and tumor tissues were obtained from the harmonized TCGA pan-cancer cohort via UCSC Xena (https://xenabrowser.net/, accessed on 3 August 2025) [18]. *ZNF668* protein expression levels were retrieved from the CPTAC module on the cProSite (https://cprosite.ccr.cancer.gov//, accessed on 3 August 2025) [19], while expression data for tumor cell lines were sourced from CCLE (https://portals.broadinstitute.org/ccle//, accessed on 3 August 2025) [20]. Additionally, the subcellular localization of *ZNF668* was determined by analyzing immunofluorescence images of three cancer cell lines (U-251MG, A431, and U2OS) from the Human Protein Atlas (https://www.proteinatlas.org//, accessed on 3 August 2025) [21].

### 2.2. Diagnostic and Prognostic Significance of ZNF668

To evaluate the diagnostic value of *ZNF668* in pan-cancer, we constructed receiver operating characteristic (ROC) curves with the standard of area under the curve (AUC) > 0.7. By integrating the expression data of *ZNF668* with relevant prognostic information, including overall survival (OS), disease-specific survival (DSS), disease-free interval (DFI), and progression-free interval (PFI), we conducted Cox proportional hazards regression analysis using the coxph function from the “survival” package (Version: 3.8-3).

### 2.3. Mutation Status of ZNF668

We performed a systematic analysis of somatic mutations in the *ZNF668* gene across various cancer types using the cBioPortal database (https://www.cbioportal.org/) [22]. Our analysis focused on assessing the frequency, type, and distribution of these mutations along the protein sequence. A lollipop plot was generated to visualize the precise location and recurrence frequency of each mutation on the *ZNF668* protein, detailing their positions relative to annotated functional domains.

### 2.4. Copy Number Variation (CNV) Analysis of ZNF668

We performed a pan-cancer analysis of *ZNF668* copy number variations (CNVs) using data from the GDC Pan-Cancer Atlas (https://gdc.cancer.gov/about-data/publications/pancanatlas). CNVs were categorized into heterozygous and homozygous amplifications and deletions. To assess the functional impact of these alterations, we integrated raw CNV data with corresponding mRNA expression profiles via TCGA barcodes and correlated CNV status of *ZNF668* with its expression levels. Statistical significance was determined using *p*-values adjusted for the False Discovery Rate (FDR).

### 2.5. Single Nucleotide Variant (SNV) Analysis of ZNF668

We calculated the single nucleotide variant (SNV) percentage (frequency of harmful mutations) for each cancer type using pan-cancer somatic mutation data from the GDC Pan-Cancer Atlas. The frequency was determined by dividing the number of mutated samples by the total number of samples analyzed. Subsequently, we integrated SNV and mRNA expression data via TCGA barcodes to correlate the mutation status of *ZNF668* with its expression level, with *p*-values adjusted for the FDR.

### 2.6. Functional Enrichment and Protein–Protein Interaction (PPI) Network Analysis of ZNF668 in Cancers

The STRING (https://cn.string-db.org/) database was used to conduct the protein–protein interaction (PPI) network analysis of *ZNF668*. Based on the “Similar Gene Detection” function of the GEPIA2 database(http://gepia2.cancer-pku.cn/), the top 200 co-expressed genes showing a significant positive correlation with *ZNF668* were selected for subsequent enrichment analysis. We then employed the “clusterProfiler” R package (Version 4.6.2) to perform Gene Ontology (GO) analysis, encompassing biological processes (BP), cellular components (CC), and molecular functions (MF). Additionally, the “GSVA” R package (Version 1.46.0) was used to conduct Gene Set Variation Analysis (GSVA), calculating single-sample enrichment scores for Hallmark gene sets from MSigDB (https://www.gsea-msigdb.org/).

### 2.7. The Role of ZNF668 in the Tumor Immune Microenvironment

To profile immune cell infiltration across pan-cancer samples, we utilized four widely recognized algorithms (EPIC, MCP-counter, xCell, and CIBERSORT) implemented via the “IOBR” R package (Version 0.99.9). Subsequently, correlation analysis was employed to investigate the association between *ZNF668* gene expression and specific immune cell subpopulations. Additionally, we also analyzed the correlation of *ZNF668* gene expression with tumor mutational burden (TMB) and microsatellite instability (MSI). Furthermore, we examined the correlation of *ZNF668* expression with a panel of key immune regulators, including major histocompatibility complex (MHC) molecules, chemokines and their receptors, and various immunosuppressive and immune-activating molecules.

### 2.8. Drug Sensitivity Analysis

To identify potential inhibitors of *ZNF668*, we comprehensively analyzed drug sensitivity data obtained from the CellMiner database (https://discover.nci.nih.gov/cellminer/) [23]. Specifically, the correlation between *ZNF668* mRNA expression levels and the half-maximal inhibitory concentration (IC50) for compounds was calculated to pinpoint candidate drugs that may suppress *ZNF668* expression.

### 2.9. Docking Validation of Drug Molecule Components and ZNF668

To investigate protein-ligand interactions, we performed molecular docking simulations. The 3D structures of *ZNF668* protein were predicted using AlphaFold [24], while the corresponding small molecule structures were retrieved in SDF format from PubChem (https://pubchem.ncbi.nlm.nih.gov/). We utilized the CB-Dock2 web server for blind docking analysis (https://cadd.labshare.cn/cb-dock2/php/blinddock.php). The binding affinity was evaluated by the Vina score, where a more negative score indicates a stronger predicted binding between the ligand and receptor.

### 2.10. Immunohistochemistry (IHC) and Evaluation

IHC was performed to evaluate the protein expression of *ZNF668* on a commercial multi-tumor tissue microarray (TMA) (Cat# ZL-MTU122; Wellbio Biotechnology, Shanghai, China), containing 20 tumor types with two 1.5 mm cores each. Briefly, after deparaffinization and rehydration, slides underwent high-pressure heat-induced epitope retrieval in EDTA buffer (pH 9.0) for 1.5 min. Endogenous peroxidase was quenched with 3% H_2_O_2_, and non-specific sites were blocked with 10% normal goat serum (Boster, Wuhan, China, AR1009). Sections were incubated overnight at 4 °C with a rabbit anti-*ZNF668* primary antibody (1:300; Bioworld, St. Louis Park, MN, USA, BS62290), followed by an HRP-conjugated goat anti-rabbit secondary antibody (1:2000; Abcam, Cambridge, UK, ab205718) for 45 min at 37 °C. The signal was developed using a DAB kit (Maxim, Fuzhou, China, DAB-4033) and sections were counterstained with Harris hematoxylin. Finally, slides were dehydrated, mounted, and digitized using a NanoZoomer^®^ S360 scanner (Hamamatsu Photonics, Hamamatsu, Japan). *ZNF668* expression was semi-quantitatively evaluated using the H-score method and the results were plotted using the ggplot2 package (Version 4.0.0) in R.

### 2.11. Statistical Analysis

All statistical analyses were conducted using R software (version 4.2.1; R Core Team, Vienna, Austria). Continuous variables were analyzed using either the *t*-test or the Wilcoxon rank-sum test, while categorical variables were compared using either the chi-squared test or Fisher’s exact test. Associations were assessed using the non-parametric Spearman’s rank correlation test. Statistical significance was defined as a *p* value < 0.05.

## 3. Results

### 3.1. ZNF668 mRNA and Protein Expression in Pan-Cancer

**Figure 1:**
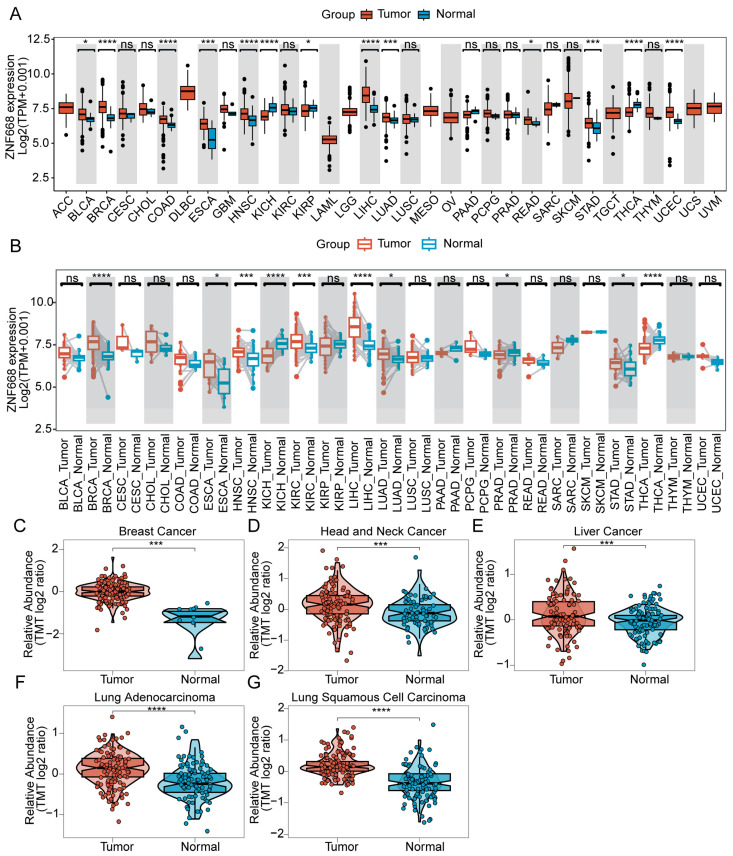
Differential expression of *ZNF668* across various cancer types. (**A**) Comparison of *ZNF668* mRNA expression between tumor and normal tissues from TCGA. (**B**) Paired analysis of *ZNF668* mRNA expression in tumor and adjacent normal tissues from TCGA. (**C**–**G**) Violin plots comparing ZNF668 protein abundance in tumor versus normal tissues from the CPTAC database for (**C**) Breast Cancer, (**D**) Head and Neck Cancer, (**E**) Liver Cancer, (**F**) Lung Adenocarcinoma, and (**G**) Lung Squamous Cell Carcinoma. Each dot represents an individual sample. ns, *p* ≥ 0.05; * *p* < 0.05; *** *p* < 0.001; **** *p* < 0.0001.

TCGA pan-cancer analysis indicated that *ZNF668* is generally highly expressed in the majority of cancer types (Figure 1A). Specifically, its mRNA expression was significantly upregulated in 10 cancer types, including BLCA, BRCA, COAD, ESCA, HNSC, LIHC, LUAD, READ, STAD, and UCEC. Conversely, *ZNF668* expression was significantly downregulated in 3 cancer types: KICH, KIRP, and THCA (*p* < 0.05). In paired samples, *ZNF668* mRNA expression was significantly upregulated in 7 cancer types: BRCA, ESCA, HNSC, KIRC, LIHC, LUAD, and STAD, while it remained significantly downregulated in KICH, PRAD, and THCA (Figure 1B). To validate these findings at the protein level, we analyzed *ZNF668* protein expression using the CPTAC database. Among the 9 cancer types with proteomics data recorded in CPTAC, *ZNF668* protein expression was significantly upregulated in 5 cancers: breast cancer, head and neck cell carcinoma, hepatocellular carcinoma, LUAD, and LUSC (Figure 1C).

### 3.2. Expression Profiles and Subcellular Localization of ZNF668 in Pan-Cancer Cell Lines

**Figure 2:**
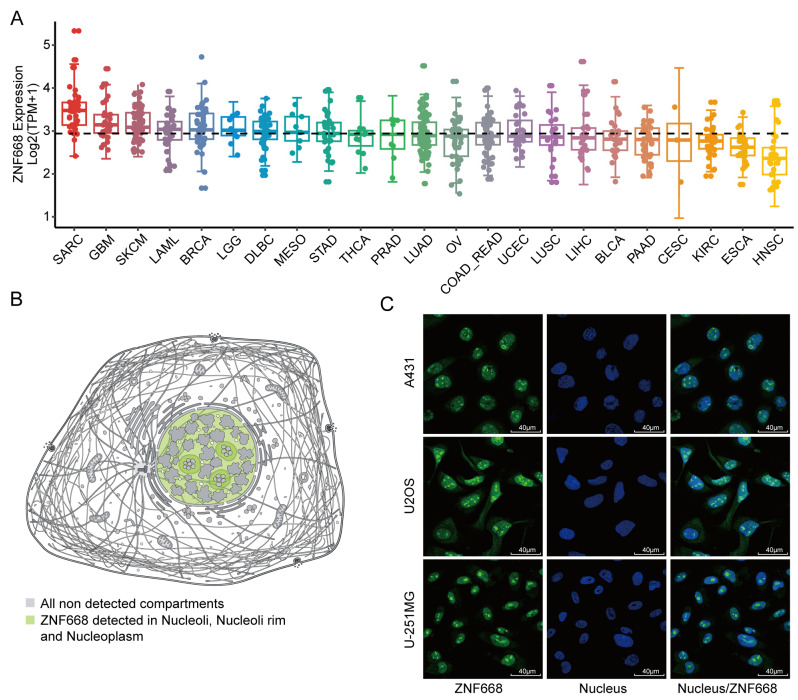
Expression and subcellular localization of *ZNF668* in cancer cell lines. (**A**) *ZNF668* mRNA expression in multiple cell lines. (**B**) Predicted subcellular localization of *ZNF668*. (**C**) Immunofluorescence images of *ZNF668* subcellular localization in tumor cells from HPA. Image credit: Human Protein Atlas. Image available from v24.0.proteinatlas.org/ENSG00000167394-*ZNF668*/subcellular.

*ZNF668* mRNA expression was analyzed across a wide range of cancer cell lines using the CCLE database. The results revealed significant heterogeneity and tissue specificity in its expression pattern (Figure 2A). The top three tumor cell lines showing the highest expression were SARC, GBM, and SKCM. In contrast, *ZNF668* expression was generally lower in various cancer cell lines of epithelial origin, with the lowest levels observed in HNSC, ESCA, and KIRC. Furthermore, the predicted subcellular localization of *ZNF668* was in the nucleus, and this was consistent with immunofluorescence validation images obtained from the HPA database. (Figure 2B,C).

### 3.3. Diagnostic Performance of ZNF668

**Figure 3:**
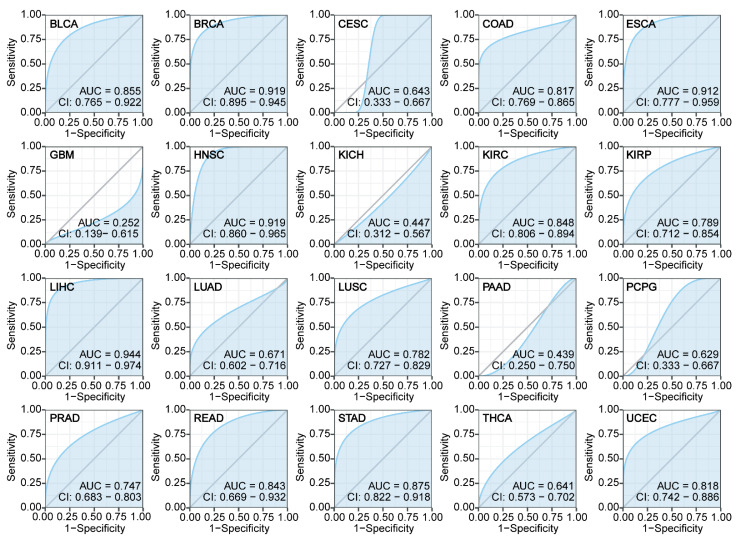
Diagnostic potential of *ZNF668* expression in pan-cancer. ROC curves illustrating the diagnostic efficacy of *ZNF668* expression across various cancer types. The grey line represents the line of no-discrimination (AUC = 0.5).

To evaluate the utility of *ZNF668* as a potential pan-cancer diagnostic biomarker, we performed ROC curve analysis of its expression levels. The results demonstrated that *ZNF668* exhibited high accuracy in distinguishing tumor tissues from normal tissues, showing excellent diagnostic performance in 13 different cancer types with an AUC greater than 0.7 (Figure 3). Specifically, these cancers included BLCA, BRCA, COAD, ESCA, HNSC, KIRC, KIRP, LIHC, LUSC, PRAD, READ, STAD, and UCEC. It is particularly noteworthy that the diagnostic predictive ability of *ZNF668* for BRCA, ESCA, HNSC, and LIHC was especially outstanding (AUC > 0.9).

### 3.4. Prognostic Significance of ZNF668 Expression

**Figure 4:**
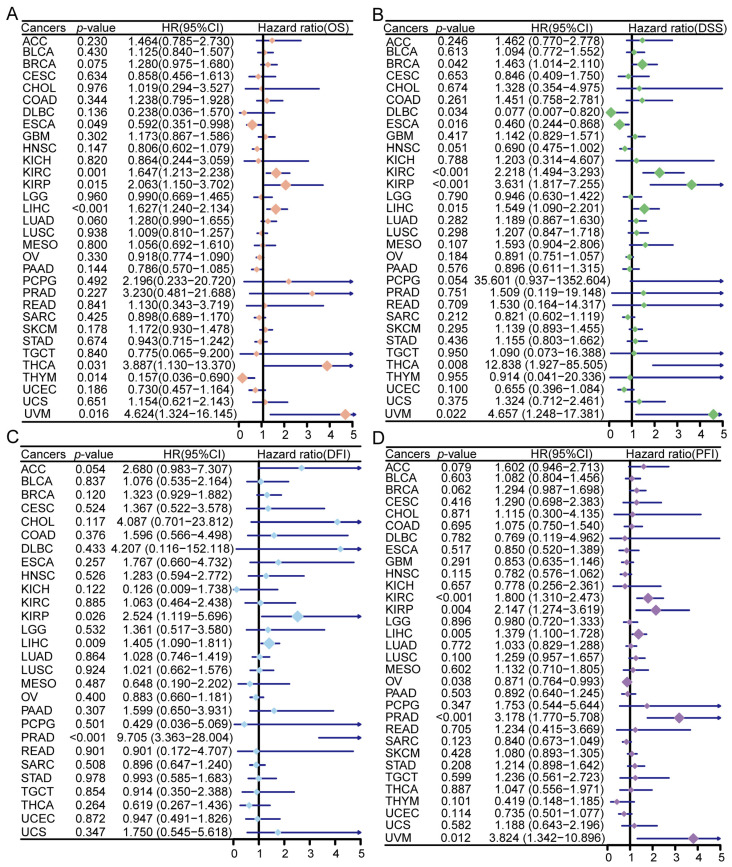
Prognostic significance of *ZNF668* expression in pan-cancer. Forest plots of univariate Cox regression analysis of *ZNF668* expression with (**A**) OS, (**B**) DSS, (**C**) DFI, and (**D**) PFI across various cancer types. The size of the diamond indicates statistical significance, with larger diamonds representing a significant result.

High *ZNF668* expression was significantly associated with OS in multiple cancer types (Figure 4A). Specifically, high *ZNF668* expression predicted a poorer OS in 5 cancer types: KIRC, KIRP, LIHC, THCA, and UVM, whereas it was associated with a better OS in ESCA and THYM. In terms of DSS, high *ZNF668* expression indicated a poor prognosis in 6 cancer types: BRCA, KIRC, KIRP, LIHC, THCA, and UVM, but predicted a better prognosis in DLBC and ESCA (Figure 4B). For DFI, elevated *ZNF668* expression was significantly correlated with a worse prognosis in KIRP, LIHC, and PRAD (Figure 4C). In the PFI analysis, high *ZNF668* expression predicted a poorer prognosis for 5 cancer types: KIRC, KIRP, LIHC, PRAD, and UVM, while it was associated with a better prognosis in OV (Figure 4D). These results suggest that *ZNF668* can serve as a potential tumor-type specific prognostic biomarker, although its significance in some cancers (e.g., DLBC, THCA, THYM) was not independent of clinical covariates in subsequent multivariate Cox analyses (Table S1).

### 3.5. Genetic Alteration Features of ZNF668

**Figure 5:**
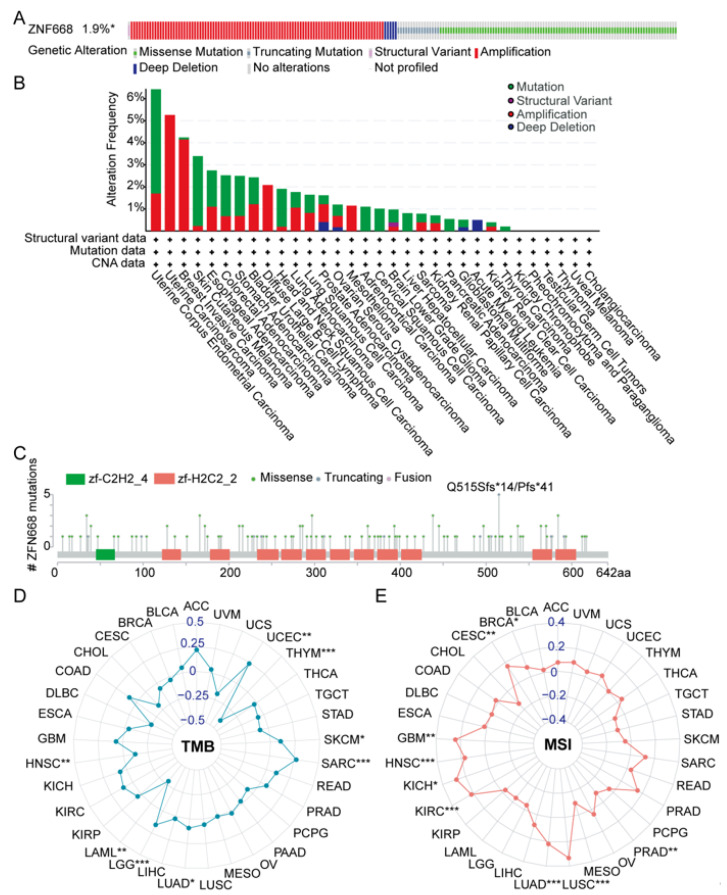
Genetic alterations of *ZNF668* and its correlation with TMB and MSI. (**A**) Summary of *ZNF668* genetic alterations (including mutations, copy number alterations, and structural variants) across various cancer types from cBioPortal. (**B**) Landscape of *ZNF668* alterations, summarizing mutation types, counts, and frequencies in different cancer types within the TCGA pan-cancer cohort. (**C**) Lollipop plot depicting the types, numbers, and specific locations of *ZNF668* somatic mutations within its protein domains in the TCGA pan-cancer cohort. (**D**) Radar plot illustrating the correlation between *ZNF668* expression and TMB across cancer types. (**E**) Radar plot illustrating the correlation between *ZNF668* expression and MSI across cancer types. * *p* < 0.05; ** *p* < 0.01; *** *p* < 0.001.

Analysis of the cBioPortal pan-cancer cohort revealed that *ZNF668* is genetically altered in 1.9% of all tumors (Figure 5A). We further examined the pan-cancer genetic alterations of *ZNF668*. UCEC, UCS, and BRCA exhibited the highest mutation rates, at 6.43%, 5.26%, and 4.24%, respectively (Figure 5B). In most cancer types, mutations or amplifications were the predominant types of genetic alterations, while structural variants and deep deletions were relatively less common. The *ZNF668* gene encodes a protein of 619 amino acids containing multiple zinc finger domains. Focusing specifically on somatic mutations, their overall prevalence was 1.0%. Among the identified *ZNF668* gene variants, a total of 123 variants of unknown significance were detected, including 106 missense mutations, 16 truncating mutations, and 1 fusion variant. Figure 5C illustrates 5 representative mutation events and their patient origins, with the truncating mutation at amino acid position 515 (Q515Sfs14/Pfs41) being relatively frequent among the mutations shown.

Prompted by this evidence of genetic instability, we correlated *ZNF668* expression with TMB and MSI. Our findings revealed varying correlations between *ZNF668* expression and TMB across different cancer types (Figure 5D). A significant positive correlation was observed in LUAD, SKCM, SARC, UCEC and LGG. Conversely, *ZNF668* expression exhibited a significant negative correlation with TMB in HNSC, LAML, and THYM. Notably, a consistent positive correlation was observed between *ZNF668* expression and MSI in several cancer types, including BRCA, CESC, GBM, HNSC, KICH, KIRC, LUAD, LUSC, and PRAD (Figure 5E).

### 3.6. Landscape of Genetic Alterations in ZNF668

**Figure 6:**
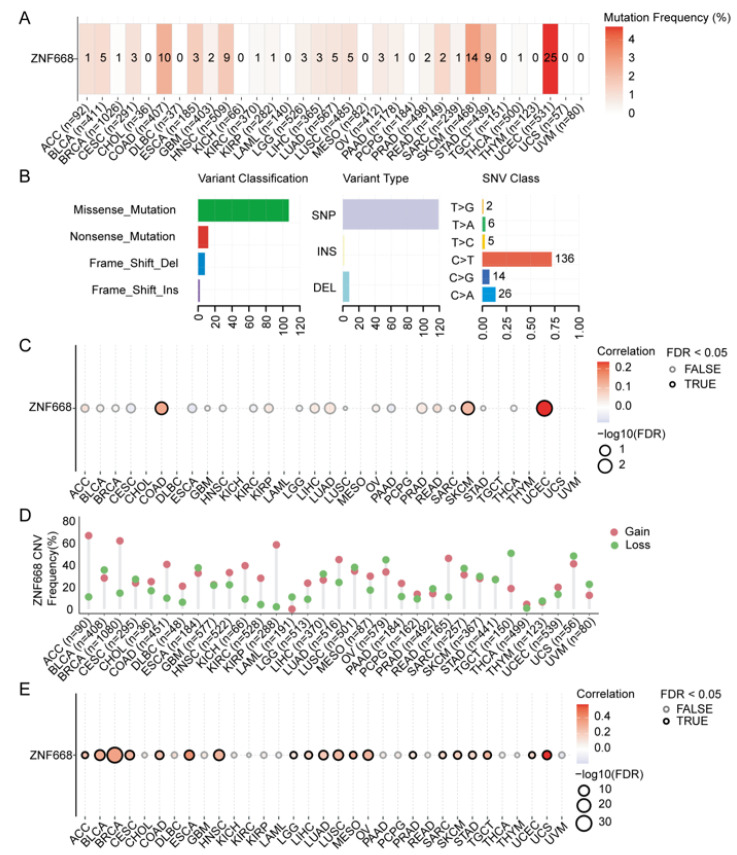
Pan-cancer genetic alteration landscape of *ZNF668*. (**A**) SNV frequencies of *ZNF668*. (**B**) Classification and mutational spectrum of *ZNF668* SNV. (**C**) Correlation of *ZNF668* SNV status with mRNA expression. (**D**) CNV levels of *ZNF668*. (**E**) Correlation of *ZNF668* CNV levels with mRNA expression.

Analysis of SNVs revealed that UCEC exhibited the highest mutation frequency (~4.7%) in the *ZNF668* gene, followed by SKCM and COAD (Figure 6A). Missense mutations were the predominant SNV type, and C-to-T transitions were the primary form of single nucleotide mutations (Figure 6B). Notably, in COAD, SKCM, and UCEC, a positive correlation was observed between *ZNF668* SNVs and its mRNA expression (Figure 6C). For CNVs, both amplifications and deletions of *ZNF668* were observed in most cancer types (Figure 6D). Among them, high-frequency homozygous amplifications were present in ACC, BRCA, and KIRP, while high-frequency heterozygous deletions were found in OV, TGCT, and UCS. Furthermore, in the majority of cancer types, a positive correlation was found between *ZNF668* CNV and its mRNA expression (Figure 6E).

### 3.7. ZNF668 Interaction Network and Functional Enrichment Analysis

**Figure 7:**
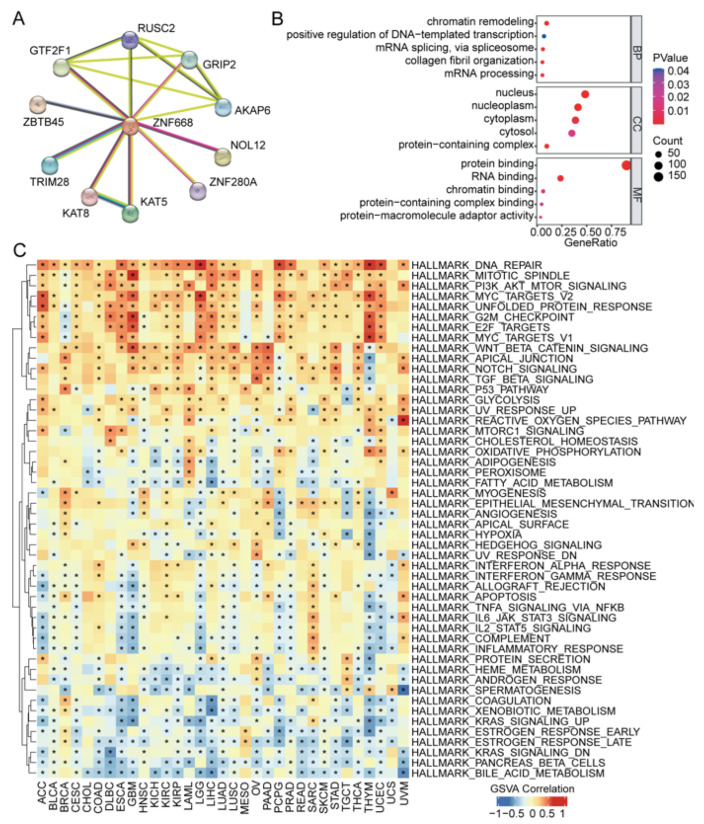
PPI networks and functional enrichment analysis of *ZNF668*. (**A**) *ZNF668*-centric PPI network. (**B**) GO term enrichment. (**C**) GSVA pathway enrichment. * *p* < 0.05.

To elucidate the functional context of *ZNF668*, we constructed a *ZNF668*-centric PPI network using the STRING database, highlighting its top 10 core interactors (Figure 7A). Subsequently, GO analysis (Figure 7B) showed that in terms of BP, genes co-expressed with *ZNF668* were significantly enriched in transcriptional regulation (e.g., “regulation of transcription by RNA polymerase II,” “chromatin remodeling”) and embryonic development. For CC, they were mainly enriched in the nucleus. At the MF level, they were primarily involved in molecular binding functions such as protein, RNA, and metal ion binding. GSVA revealed a robust positive correlation with pathways integral to cell proliferation and survival, including DNA repair, mitotic spindle, G2M checkpoint, *PI3K/AKT/MTOR* signaling, myc targets, and the unfolded protein response. Conversely, its expression was significantly and negatively correlated with pathways associated with specialized metabolism and signaling pathways, such as xenobiotic metabolism, bile acid metabolism, *KRAS* signaling, and estrogen response (Figure 7C).

### 3.8. Association of ZNF668 Expression with Immune Regulatory Molecules

**Figure 8:**
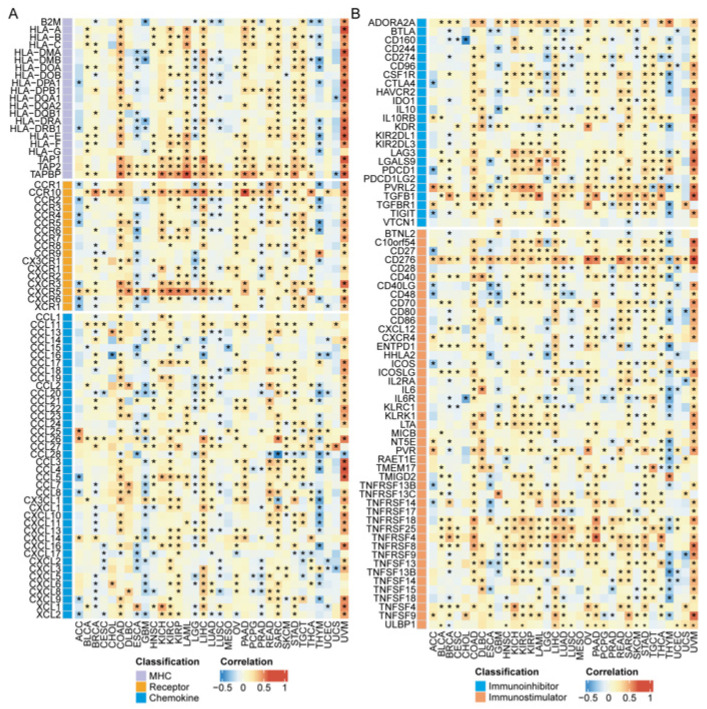
Pan-cancer correlation analysis between *ZNF668* expression and immune regulatory molecules. (**A**) Heatmap illustrating the correlation coefficients between *ZNF668* expression and MHC molecules, chemokines, and chemokine receptors across various cancer types. (**B**) Heatmap illustrating the correlation coefficients between *ZNF668* expression and immunoinhibitors and immunostimulators across various cancer types. * *p* < 0.05.

*ZNF668* expression exhibited a remarkably consistent positive correlation with a large number of immune regulatory genes across diverse cancer types. This pattern was particularly strong for key costimulatory and coinhibitory molecules. For instance, strong positive associations were consistently observed for antigen presentation machinery components like *TAP1*, *TAP2*, and *TAPBP* (Figure 8A). Similarly, *ZNF668* was positively correlated with *PVRL2 (Nectin-2)*, *CD276 (B7-H3)*, *TGFB1*, and multiple members of the TNF receptor superfamily, including *TNFRSF4 (OX40)*, *TNFRSF18 (GITR)*, and *TNFRSF25*, in the vast majority of analyzed cancers (Figure 8B). Other key immune molecules that followed this predominantly positive correlation pattern include the immune checkpoint regulators *ADORA2A*, *CTLA4*, *LAG3*, and *PDCD1 (PD-1)*, as well as the costimulatory molecules *CD27*, *ICOSLG*, and *LTA* (Figure 8B). This widespread positive association extended to crucial chemokine receptors such as *CXCR5* and *CCR10*, which were significantly correlated with *ZNF668* in over 15 distinct malignancies (Figure 8A).

In contrast to the widespread positive associations, the correlation of *ZNF668* with a subset of immune genes was heterogeneous, varying significantly between different cancer types. The association with *B2M* exemplifies this pattern, showing a positive correlation in tumors like COAD and PCPG, but a significant negative correlation in GBM, LUAD, LUSC, and THCA. This heterogeneity was particularly evident among major immune checkpoints and costimulatory molecules (Figure 8A). For example, *CD274 (PD-L1)* and *TIGIT* were positively correlated with *ZNF668* in gastrointestinal cancers (COAD, STAD, LIHC) but were negatively correlated in others, such as BRCA, LGG, and LUSC (Figure 8B). Similarly, the costimulatory molecule *CD86* showed a positive correlation in COAD and LIHC but a strong negative correlation in GBM, LGG, and LUSC (Figure 8B). This context-dependency also extended to molecules like *TNFRSF14 (HVEM)*, *CSF1R*, and several HLA genes, which were positively correlated in cancers like COAD and LIHC but negatively correlated in ESCA, LGG, and LUSC (Figure 8A,B). While a consistent negative correlation with a single gene across most cancers was rare, it was a defining feature for a few chemokines, most notably *CXCL17* and *CCL28*. Furthermore, we identified a consistent negative correlation between *ZNF668* expression and a specific group of chemokines primarily responsible for neutrophil recruitment, notably *CXCL8*, *CXCL2*, and *CXCL5* (Figure 8A).

### 3.9. Correlation of ZNF668 Expression with Immune Cell Infiltration

**Figure 9:**
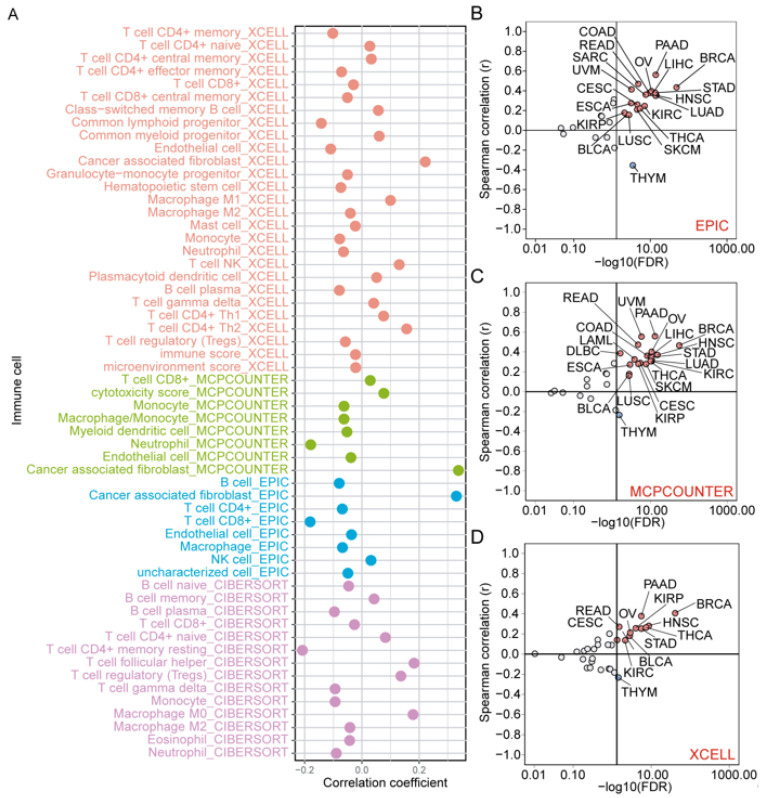
Pan-Cancer analysis of *ZNF668* expression and immune cell infiltration. (**A**) Correlations between *ZNF668* expression and various immune cell subtypes. Correlation analysis between ZNF668 expression and CAF infiltration across pan-cancer cohorts, calculated using the (**B**) EPIC, (**C**) MCP-counter, and (**D**) xCell algorithms. Dots represent cancer types. Color indicates correlation significance (FDR < 0.05): red, positive; blue, negative; white, non-significant.

*ZNF668* expression broadly correlated with immune cell infiltration across various cancers. Notably, among these correlations, *ZNF668* expression showed a consistent and significant positive correlation with the infiltration of CAFs, confirmed by integrating results from three distinct computational algorithms: EPIC, MCP-counter, and xCell (Figure 9A). Further analysis revealed a strong and consistent positive correlation between *ZNF668* expression levels and CAF abundance in the vast majority of analyzed cancer types, such as PAAD, READ, and BRCA (Figure 9B–D). In sharp contrast, *ZNF668* expression showed a consistent negative correlation with CAF infiltration in THYM.

### 3.10. Drug Sensitivity Analysis and Molecular Docking

**Figure 10:**
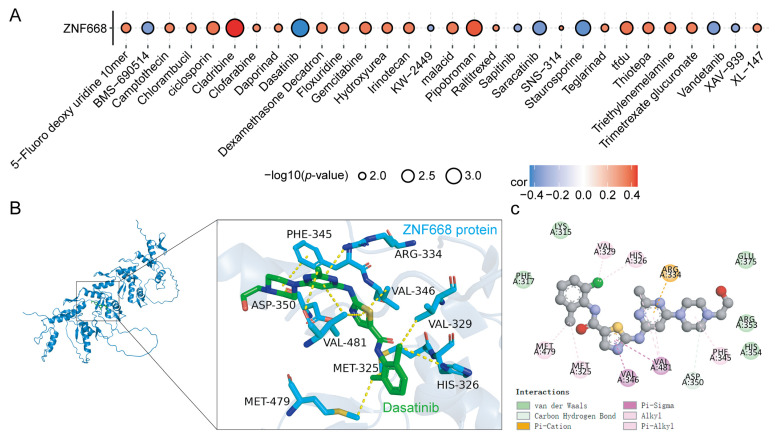
Drug sensitivity analysis for *ZNF668*. (**A**) Correlation of *ZNF668* mRNA expression with drug response (IC50) from the CellMiner database. (**B**) 3D binding pose of Dasatinib interacting with *ZNF668* protein. (**C**) 2D interaction diagram showing detailed interactions between Dasatinib and *ZNF668* protein residues.

To identify potential inhibitors targeting *ZNF668*, we evaluated the correlation between *ZNF668* mRNA expression and drug sensitivity data obtained from the CellMiner database. Among all screened agents, Dasatinib exhibited the strongest negative correlation with *ZNF668* expression (rho= −0.441, *p* < 0.05) (Figure 10A), suggesting a hypothesis that Dasatinib might exert its effects by potentially interacting with *ZNF668* for anti-tumor effects. Molecular docking further demonstrated that Dasatinib stably binds to *ZNF668*, with a low binding energy of −7.6 kcal/mol, reflecting the high stability of the resulting complex (Figure 10B). The complex is stabilized through hydrogen bonds with PHE-345 and ASP-350, a π-cation interaction with ARG-334, and hydrophobic contributions from MET-325, MET-479, VAL-329, VAL-346, VAL-481, and HIS-326 (Figure 10C).

### 3.11. Validation of ZNF668 Expression by TMA

**Figure 11:**
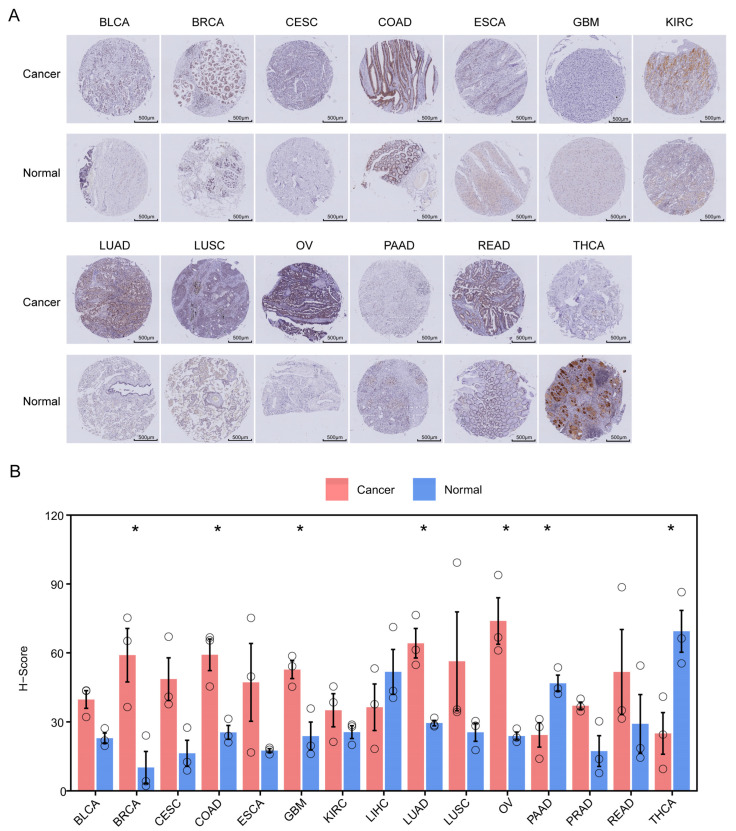
IHC analysis of *ZNF668* protein expression in pan-cancer tissues. (**A**) IHC staining for *ZNF668* in various cancer and normal tissues. (**B**) Quantification of *ZNF668* expression using H-scores. Data are shown as mean ± SEM. The circle represents an individual sample. * *p* < 0.05.

Corroborating our bioinformatic predictions, IHC results confirmed that *ZNF668* protein levels were significantly elevated in BRCA, COAD, GBM, LUAD, and OV, while conversely being significantly decreased in PAAD and THCA (Figure 11A,B).

## 4. Discussion

This study provides a comprehensive pan-cancer analysis of *ZNF668*, investigating its expression patterns, prognostic value, genomic alterations, functional pathways, and its complex interplay with the tumor immune microenvironment. Furthermore, to explore its therapeutic potential, we employed drug sensitivity correlation and molecular docking to identify potential inhibitors.

We first established the clinical relevance of *ZNF668* by analyzing its expression and prognostic value across a wide range of human cancers. *ZNF668* was frequently dysregulated, exhibiting prominent overexpression in malignancies such as BRCA, HNSC, LIHC, and LUAD, while showing reduced expression in others, including KICH and THCA. Subsequently, our IHC analysis served to both validate and expand upon our bioinformatic findings. We confirmed the significant *ZNF668* upregulation in BRCA and LUAD and downregulation in THCA, which aligned perfectly with the TCGA and CPTAC database analyses. Furthermore, we supplemented the database findings by providing the protein-level evidence of significant *ZNF668* overexpression in OV. However, while our IHC showed *ZNF668* was significantly upregulated in COAD and GBM, in contrast to the non-significant results from TCGA, the trend of upregulation was consistent.

Beyond its dysregulated expression, *ZNF668* showed significant potential as a clinical biomarker, underscored by its high diagnostic accuracy for distinguishing tumors from normal tissue (AUC > 0.9 in BRCA, ESCA, HNSC, and LIHC) and its consistent association with shorter OS, DSS, and PFI in KIRP and LIHC. However, these clinical findings present an apparent paradox when contrasted with previous in vitro research. While the oncogenic role aligns with reports of *ZNF668* promoting proliferation in leukemia cells [25], it contradicts studies where *ZNF668* acts as a potent inhibitor of migration and invasion in lung and bladder cancer cells [26,27]. This raises the question of why *ZNF668* functions as a tumor suppressor in isolated epithelial cancer cells but correlates with poor prognosis in patient tumors of the same origin.

To resolve this discrepancy, our analysis of the CCLE database pointed towards a context-dependent role for *ZNF668*, as we found its expression was significantly higher in cell lines of mesenchymal origin (e.g., SARC, GBM, LAML) compared to those from many epithelial cancers (e.g., HNSC, ESCA). This suggested a lineage-specific functional switch, wherein it may act as an oncogene in high-expression mesenchymal tumors but as a tumor suppressor in low-expression epithelial cancers. However, our clinical findings challenge this hypothesis, as *ZNF668* overexpression in epithelial tumors like LIHC consistently predicts poor survival. This discrepancy reveals the limitations of cell-line models and points to the TME as a critical determinant of *ZNF668*’s ultimate oncogenic role.

Further analysis revealed that a high-*ZNF668* TME presents a concurrently inflamed yet profoundly immunosuppressive state. Specifically, the TME is immunologically “hot,” as *ZNF668*’s positive correlation with T-cell co-stimulatory molecules (e.g., *TNFRSF4*, *CD27*) and antigen presentation machinery (e.g., *TAP1*, *TAPBP*) indicates T-cell recruitment and activation [28,29]. However, this is counterbalanced by marked immunosuppression, characterized by upregulated checkpoints like *PD-1*, *CTLA4*, and *LAG3*. This paradox may be mechanistically linked to a strong positive correlation between *ZNF668* and CAF abundance. CAFs, through intricate signaling networks often involving TGF-β, are known to drive treatment failure and immune escape [30,31,32]. We thus propose that a CAF-driven, TGF-β-mediated suppressive network underlies this state of adaptive immune resistance, resulting in a T-cell-infiltrated but functionally exhausted TME [33].

While the *ZNF668*-CAF axis suggests a significant mechanism for immune evasion, its immunomodulatory influence also extends to other aspects of TME. We found that *ZNF668* expression exhibited a consistent positive correlation with MSI across cancers. Since MSI is a hallmark of defective DNA mismatch repair (MMR) that results in a hypermutated phenotype and abundant immunogenic neoantigens [34,35], this finding suggests that high *ZNF668* expression may either contribute to this MSI-high phenotype or be associated with impaired MMR function. Moreover, we observed that *ZNF668* expression was consistently negatively correlated with a specific group of pro-inflammatory chemokines (notably *CXCL8*, *CXCL2*, and *CXCL5*) that are primarily responsible for neutrophil recruitment [36,37]. Despite the above consistent associations, *ZNF668*’s role exhibits significant context-dependency and complexity at a broader level. For instance, its association with key molecules like *CD274 (PD-L1)* and *B2M* is positive in gastrointestinal cancers but shifts to a negative correlation in others, including BRCA, LUSC, and GBM. This heterogeneity also extends to TMB, which is positively correlated with *ZNF668* in LUAD and SKCM yet negatively correlated in HNSC and LAML.

GO enrichment analysis revealed that *ZNF668* is predominantly enriched in fundamental nuclear processes, including the spliceosome, transcriptional regulation, and chromatin remodeling. These pathways are integral to the establishment and maintenance of malignant phenotypes. The spliceosome, for instance, contributes to oncogenesis not only through its canonical alternative splicing activities that reshape the tumor transcriptome and proteome, but also via non-canonical, splicing-independent mechanisms that influence a wide range of core aspects of cancer progression [38,39]. Dysregulation of transcriptional control reshapes gene expression programs to drive tumor progression [40], while imbalances in chromatin remodeling regulate gene expression and exacerbate splicing abnormalities by altering chromatin accessibility and histone modifications [41]. The functional consequence of this regulation is evident in GSVA, which revealed that its expression was positively correlated with pathways integral to cell proliferation and survival. The convergence of these functions within the nucleus is highly consistent with the established nuclear localization of the *ZNF668* protein, reinforcing the hypothesis that it functions as a critical upstream regulatory hub governing key oncogenic processes.

We also investigated the upstream mechanisms of the aberrant expression of *ZNF668* by analyzing its genetic alteration features. Amplification is the most predominant type of genetic alteration for *ZNF668* in multiple cancers, and its mRNA expression level is significantly positively correlated with its copy number variation level in the vast majority of tumors. This provides a potential molecular explanation for the abnormal expression of *ZNF668* and is consistent with the mechanism by which many known oncogenes promote tumorigenesis through copy number amplification [42].

Given the strong association between *ZNF668* and CAF abundance revealed by our bioinformatic analysis, we sought to identify its potential inhibitors. Through an analysis of drug sensitivity profiles, we discovered a strong negative correlation between *ZNF668* expression and response to Dasatinib. This association was further supported by preliminary molecular docking simulations, which indicated a potential stable binding between the Dasatinib and the *ZNF668* protein. Dasatinib, a multi-kinase inhibitor targeting BCR-ABL, *SRC*, and c-*KIT*, has been shown not only to inhibit the growth of lung cancer-derived CAFs but also to revert their oncogenic phenotype to that of non-tumorigenic fibroblasts [43]. Furthermore, Dasatinib enhances immunotherapy efficacy by reprogramming CAFs. In models of colorectal and triple-negative breast cancer, Dasatinib selectively inhibits CAFs and remodels the extracellular matrix, thereby promoting the deep infiltration of drugs and CD8+ T cells into the tumor, converting immunologically “cold” tumors into “hot” tumors and significantly enhancing the efficacy of anti-PD-1 immunotherapy [44,45]. While these potent CAF-inhibitory effects of Dasatinib are well-documented, its molecular target remains unclear. Our findings provide a novel mechanistic link between these independent observations, leading us to propose the scientific hypothesis that *ZNF668* may be the key molecular mediator through which Dasatinib exerts its CAF-modulating and immune-reprogramming functions. This hypothesis warrants further experimental investigation.

This study has certain limitations. The discrepant associations observed across different tumor types underscore the context-dependent functionality of *ZNF668*, warranting further investigation to delineate its specific mechanistic roles. Furthermore, while *ZNF668* shows promise as a biomarker, its prognostic and diagnostic utility requires robust validation in larger, prospectively collected clinical cohorts. Critically, the proposition of Dasatinib as a putative inhibitor of *ZNF668* is currently based solely on in silico predictions; therefore, empirical corroboration via biophysical assays, such as the cellular thermal shift assay or surface plasmon resonance, is required to confirm a direct interaction.

## 5. Conclusions

In summary, this study redefines *ZNF668* as a key mediator of immunosuppression and a biomarker for poor prognosis. Our work reconciles the conflicting reports of *ZNF668*’s tumor-suppressive in vitro activities with its clear pro-tumorigenic association in patients, identifying the TME as the decisive, context-dependent switch, which is mechanistically underscored by a strong correlation with CAF abundance that cultivates a T-cell-exhausted, immunosuppressive landscape. This association provides a clear rationale for targeting *ZNF668*, with Dasatinib identified as a potential inhibitor. Our study provides a new framework for restoring effective anti-tumor immunity by targeting *ZNF668* to dismantle the immunosuppressive TME.

## Data Availability

The original data presented in the study are openly available in TCGA and other databases. The raw data supporting the conclusions of this article will be made available by the corresponding authors on request.

## Supplementary Materials

The following supporting information can be downloaded at: https://www.mdpi.com/article/10.3390/cimb47120997/s1.
